# Ageing in patients with chronic HIV infection: impact of hypercoagulation

**DOI:** 10.1186/s12981-018-0211-1

**Published:** 2018-11-24

**Authors:** Stephen J. Kent, Charles Flexner

**Affiliations:** 1 Department of Microbiology and Immunology, Peter Doherty Institute for Infection and Immunity, University of Melbourne, Parkville, VIC 3010 Australia; 2 Melbourne Sexual Health Centre and Department of Infectious Diseases, Alfred Health, Central Clinical School, Monash University, Melbourne, Australia; 3 ARC Centre of Excellence in Convergent Bio-Nano Science and Technology, University of Melbourne, Parkville, Australia; 4 Divisions of Clinical Pharmacology and Infectious Diseases, School of Medicine and Bloomberg School of Public Health, Johns Hopkins University, Baltimore, MD USA

**Keywords:** HIV, Ageing, Fraility, Antiretroviral therapy, Coagulation, Inflammation

## Abstract

Ageing is the result of biological events that progressively and irreversibly compromise the function of vital organs and eventually result in death. There is a general perception that ageing is accelerated in people living with HIV, with an increasing body of evidence to support this view. With the introduction of effective antiretroviral therapy, the life expectancy of people living with HIV has improved. Since people with HIV are living longer than previously, while also ageing faster than the general population, there is an increase in HIV-positive patients living with age-related comorbidities. This brief overview of ageing and HIV discusses aspects of the complications of HIV infection as they impact the ageing process. How diseases of age affect patients with HIV provides clues to help unravel the interactions between HIV and ageing that ultimately should help clinicians understand the basis of ‘normal’ ageing and manage ageing HIV-positive patients more effectively.

## Background

In the past decade, there has been a dramatic improvement in the life expectancy of people living with HIV, as a result of effective antiretroviral therapy (ART) [1]. This has led to an increase in the average age of HIV-positive patients, which presents new clinical issues with regard to ageing and the associated comorbidities [2].

To date, the oldest known human lived for 122 years, but life expectancy in most developed countries is 80–85 years. However, even with recent improvements in the treatment of HIV, life expectancy for people living with HIV is well below that of the general population [2].

The causes and limits of ‘normal’ human ageing are poorly understood. Defining the process, understanding the mechanisms, and using this knowledge to slow or reverse ageing is a field in its relative infancy. There is a general perception that HIV infection ‘accelerates’ ageing, with mounting evidence to support this view [3, 4]. A range of hypotheses exist for the presence of accelerated aging in subjects with HIV. These including reduced telomerase capacity, reduced somatic stem cell proliferation potential, and the effects of gut bacterial translocation. Aging may be also commonly seen due to lifestyle differences between HIV infected cohorts and the general population. This brief review summarizes a recent symposium presentation in this field and focusses on the impact of hypercoagulation and micro-thrombotic events as they relate to ageing and HIV infection.

## Metabolic and physiologic changes occur with age

The genetic basis of ageing is complex and no single gene or set of genes has clearly been linked to normal human ageing across the population [5, 6]. Numerous studies have identified metabolic and physiologic changes that occur with age. Creatinine clearance declines by approximately 30% between the ages of 30 and 80 [7]. Similarly, cardio-respiratory fitness, as measured physiologically and expressed in metabolic equivalents, declines by around 40% from age 30 to 80 [8]. Bone mass also declines progressively with age, at a similar rate in men and women, but men take longer to reach a fracture threshold due to starting with a higher bone mass [9].

## Ageing: the result of organ dysfunction

Rather than an inherent biological clock, the average rate at which individuals in a species age is determined by the level of organ dysfunction. This is a balance between the number and rate of dysfunction events, and the capacity of vital organs to tolerate those events.

Over time, cumulative events compromise the function of vital organs, leading to age-related disease and, eventually, death. This is linked to the concept of frailty.

## Reduced blood flow to organs causes functional decline

One important reason for the progressive and often irreversible decline in organ function that occurs with ageing is a reduction in blood flow to the organ. The rate of liver blood flow, as reflected by the half-lives of liver-metabolised drugs, is reduced with ageing [10]. Kidney blood flow, as reflected by renal perfusion, also declines with age [11]. These combined effects have been shown to influence the reduced clearance of HIV drugs, such as lopinavir, as subjects age [12].

A primary reason that blood flow to vital organs declines with age is that blood becomes more hypercoagulable. This is reflected by marked increases in average d-dimer concentrations in people over 80 years of age [13]. Thus, ageing could be driven by thrombotic events that progressively and irreversibly compromise the function of vital organs. Sir William Osler famously noted, “Longevity is a vascular question, which has been well expressed in the axiom ‘a man is as old as his arteries’. To a majority of men death comes primarily or secondarily through this portal.”

## Inflammatory markers increase with age

**Fig. 1:**
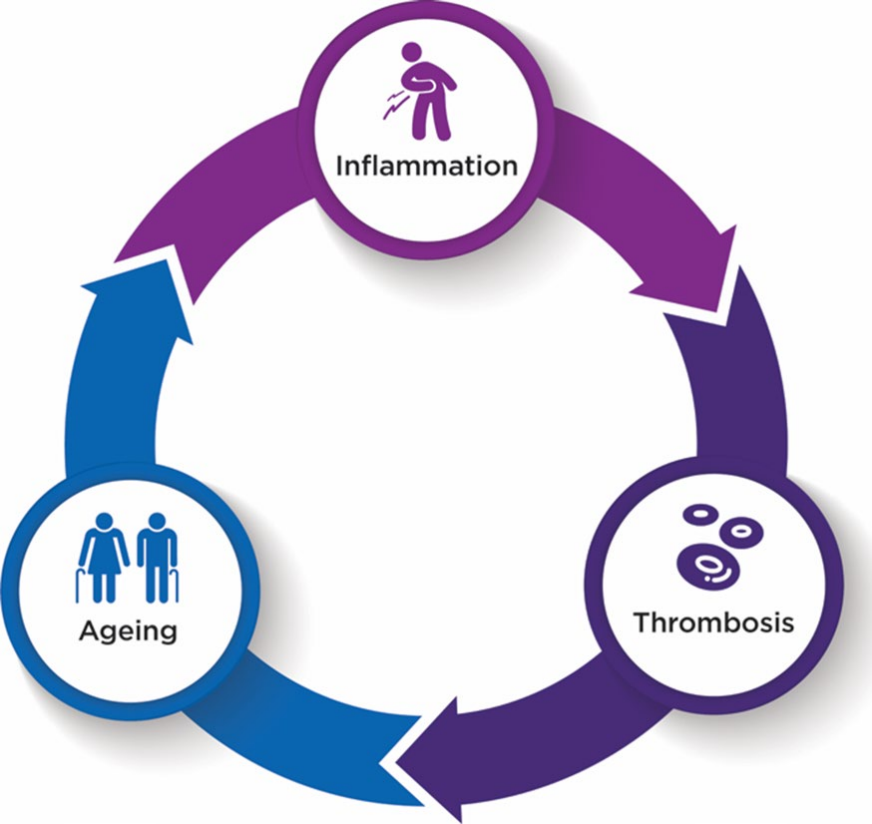
The relationship between inflammation, thrombosis and ageing. Inflammation increases with age. This leads to an increase in hypercoagulability, which compromises blood flow to organs, and subsequently drives the ageing process and further inflammation. HIV causes enhanced coagulation as measured by d-dimer assays, enhanced inflammation as measured by IL-6 and other cytokine levels and enhanced carotid intimal thickness and cardiovascular disease

Markers of inflammation such as C-reactive protein and fibrinogen show significant increases with age in otherwise healthy people [14]. Inflammatory cytokines such as interleukin 6 (IL-6) and tumour necrosis factor (TNF) increase in arterial endothelial cells with age [15]. Thus, ageing may be the consequence of cumulative inflammatory events that progressively drive thrombosis and irreversibly compromise blood flow and the function of vital organs, as illustrated in Fig. 1.

## HIV is associated with reduced life expectancy

HIV infection, even when appropriately treated with ART, is associated with at least a modestly reduced life expectancy [2]. Historically, the cause of death in people infected with HIV was opportunistic infection. Chronic diseases associated with ageing are now a leading cause of death in HIV-positive patients [16–19].

The link between hypercoagulation, inflammation and adverse outcomes in treated HIV infection has become increasingly evident [20]. In the SMART study, 5472 participants were randomised to continue treatment with their current ART or to discontinue treatment while their CD4+ count remained above 250 [21]. Episodic ART significantly increased risk of death from any cause, compared to those who remained on continuous treatment. In this cohort of HIV-positive patients, cause of death was primarily related to diseases of age, such as cardiovascular, renal or hepatic disease, not opportunistic infections [21], and has been strongly linked to increases in the coagulation and inflammatory markers d-dimer and IL-6 [22].

Cohort studies have identified that HIV-related ageing related disease is more common in the areas of heart disease, diabetes, neuropathies and thrombotic events rather than all organ systems [23]. This lends support to the micro coagulation hypothesis.

Physiological changes that occur in HIV-positive patients may be associated with age-related comorbidities. Carotid intimal thickness is increased in HIV-positive patients compared to HIV-negative patients [24]. HIV leads to a ‘leaky gut’ syndrome, where higher levels of bacterial lipopolysaccharide and other bacterial products are present in the blood. This leads to increased vascular inflammation and is widely thought to underpin much of the excess morbidity and mortality in HIV-positive patients who are otherwise well [25]. Inflammation levels are less in HIV-infected subjects virologically suppressed with ART but still abnormal and this may underlie the still substantial morbidity of HIV infection despite ART [26]. With the ageing HIV population, we might expect an increase in small-vessel ‘vascular’ dementia associated with HIV in the future, although this has not been definitively observed to date.

Cumulative toxicities from ART and associated medications are common in elderly HIV infected subjects [27]. Renal impairment is common and linked to common HIV medications [28]. Medical treatment of HIV infection and associated comorbidities such as cardiovascular and renal disease requires careful management by clinicians and dedicated pharmacists [29].

## Conclusions

HIV infection can be viewed as a model of accelerated ageing, with increased osteopenia, cardiovascular, renal and hepatic disease at a younger age [30]. The differential effect of HIV on end-organ diseases of ageing provides clues to help unravel the interactions between HIV and ageing that ultimately should help clinicians understand the basis of ‘normal’ ageing and manage ageing HIV-positive patients more effectively.

## Abbreviations

ART: antiretroviral therapy
IL-6: interleukin 6
TNF: tumour necrosis factor

## References

[1] May M, Gompels M, Delpech V, Porter K, Post F, Johnson M, Dunn D, Palfreeman A, Gilson R, Gazzard B (2011). Impact of late diagnosis and treatment on life expectancy in people with HIV-1: UK Collaborative HIV Cohort (UK CHIC) Study. BMJ.

[2] Sabin CA, Reiss P (2017). Epidemiology of ageing with HIV: what can we learn from cohorts?. AIDS.

[3] Horvath S, Levine AJ (2015). HIV-1 infection accelerates age according to the epigenetic clock. J Infect Dis.

[4] Rickabaugh TM, Baxter RM, Sehl M, Sinsheimer JS, Hultin PM, Hultin LE, Quach A, Martinez-Maza O, Horvath S, Vilain E, Jamieson BD (2015). Acceleration of age-associated methylation patterns in HIV-1-infected adults. PLoS ONE.

[5] Walter S, Atzmon G, Demerath EW, Garcia ME, Kaplan RC, Kumari M, Lunetta KL, Milaneschi Y, Tanaka T, Tranah GJ (2011). A genome-wide association study of aging. Neurobiol Aging.

[6] Lunetta KL, D’Agostino RB, Karasik D, Benjamin EJ, Guo CY, Govindaraju R, Kiel DP, Kelly-Hayes M, Massaro JM, Pencina MJ (2007). Genetic correlates of longevity and selected age-related phenotypes: a genome-wide association study in the Framingham Study. BMC Med Genet.

[7] Rowe JW, Andres R, Tobin JD, Norris AH, Shock NW (1976). The effect of age on creatinine clearance in men: a cross-sectional and longitudinal study. J Gerontol.

[8] Jackson AS, Sui X, Hebert JR, Church TS, Blair SN (2009). Role of lifestyle and aging on the longitudinal change in cardiorespiratory fitness. Arch Intern Med.

[9] Egrise D, Vienne A, Martin D, Chaboteaux C, Bergmann P, Schoutens A (1999). Age-related inhibitory activity of rat bone marrow supernatant on osteoblast proliferation. J Bone Miner Res.

[10] Sotaniemi EA, Arranto AJ, Pelkonen O, Pasanen M (1997). Age and cytochrome P450-linked drug metabolism in humans: an analysis of 226 subjects with equal histopathologic conditions. Clin Pharmacol Ther.

[11] Epstein M (1996). Aging and the kidney. J Am Soc Nephrol.

[12] Crawford KW, Spritzler J, Kalayjian RC, Parsons T, Landay A, Pollard R, Stocker V, Lederman MM, Flexner C, Team ACTP (2010). Age-related changes in plasma concentrations of the HIV protease inhibitor lopinavir. AIDS Res Hum Retroviruses.

[13] Harper PL, Theakston E, Ahmed J, Ockelford P (2007). d-dimer concentration increases with age reducing the clinical value of the d-dimer assay in the elderly. Intern Med J.

[14] Ferrucci L, Corsi A, Lauretani F, Bandinelli S, Bartali B, Taub DD, Guralnik JM, Longo DL (2005). The origins of age-related proinflammatory state. Blood.

[15] Donato AJ, Black AD, Jablonski KL, Gano LB, Seals DR (2008). Aging is associated with greater nuclear NF kappa B, reduced I kappa B alpha, and increased expression of proinflammatory cytokines in vascular endothelial cells of healthy humans. Aging Cell.

[16] Hentzien M, Drame M, Allavena C, Jacomet C, Valantin MA, Cabie A, Cuzin L, Rey D, Pugliese P, Bani-Sadr F, Dat ASG (2016). Impact of age-related comorbidities on five-year overall mortality among elderly HIV-infected patients in the late HAART era-role of chronic renal disease. J Nutr Health Aging.

[17] Smith CJ, Ryom L, Weber R, Morlat P, Pradier C, Reiss P, Kowalska JD, de Wit S, Law M, el Sadr W (2014). Trends in underlying causes of death in people with HIV from 1999 to 2011 (D:A:D): a multicohort collaboration. Lancet.

[18] Deeks SG, Lewin SR, Havlir DV (2013). The end of AIDS: HIV infection as a chronic disease. Lancet.

[19] Guaraldi G, Palella FJ (2017). Clinical implications of aging with HIV infection: perspectives and the future medical care agenda. AIDS.

[20] Lagathu C, Cossarizza A, Bereziat V, Nasi M, Capeau J, Pinti M (2017). Basic science and pathogenesis of ageing with HIV: potential mechanisms and biomarkers. AIDS.

[21] El-Sadr WM, Lundgren J, Neaton JD, Gordin F, Abrams D, Arduino RC, Babiker A, Burman W, Clumeck N, Strategies for Management of Antiretroviral Therapy Study G (2006). CD4+ count-guided interruption of antiretroviral treatment. N Engl J Med.

[22] Grund B, Baker JV, Deeks SG, Wolfson J, Wentworth D, Cozzi-Lepri A, Cohen CJ, Phillips A, Lundgren JD, Neaton JD (2016). Group ISESS: relevance of interleukin-6 and d-dimer for serious non-AIDS morbidity and death among HIV-positive adults on suppressive antiretroviral therapy. PLoS ONE.

[23] Petoumenos K, Huang R, Hoy J, Bloch M, Templeton DJ, Baker D, Giles M, Law MG, Cooper DA (2017). Prevalence of self-reported comorbidities in HIV positive and HIV negative men who have sex with men over 55 years-The Australian Positive & Peers Longevity Evaluation Study (APPLES). PLoS ONE.

[24] Hsue PY, Hunt PW, Sinclair E, Bredt B, Franklin A, Killian M, Hoh R, Martin JN, McCune JM, Waters DD, Deeks SG (2006). Increased carotid intima-media thickness in HIV patients is associated with increased cytomegalovirus-specific T-cell responses. AIDS.

[25] Brenchley JM, Price DA, Schacker TW, Asher TE, Silvestri G, Rao S, Kazzaz Z, Bornstein E, Lambotte O, Altmann D (2006). Microbial translocation is a cause of systemic immune activation in chronic HIV infection. Nat Med.

[26] Hunt PW (2017). Very early ART and persistent inflammation in treated HIV. Clin Infect Dis.

[27] Marzolini C, Back D, Weber R, Furrer H, Cavassini M, Calmy A, Vernazza P, Bernasconi E, Khoo S, Battegay M (2011). Ageing with HIV: medication use and risk for potential drug-drug interactions. J Antimicrob Chemother.

[28] Bansi L, Hughes A, Bhagani S, Mackie NE, Leen C, Levy J, Edwards S, Connolly J, Holt SG, Hendry BM (2009). Clinical epidemiology of HIV-associated end-stage renal failure in the UK. AIDS.

[29] Blaylock JM, Wortmann GW (2015). Care of the aging HIV patient. Cleve Clin J Med.

[30] Guaraldi G, Orlando G, Zona S, Menozzi M, Carli F, Garlassi E, Berti A, Rossi E, Roverato A, Palella F (2011). Premature age-related comorbidities among HIV-infected persons compared with the general population. Clin Infect Dis.

